# The Role of γδ T-Lymphocytes in Glioblastoma: Current Trends and Future Directions

**DOI:** 10.3390/cancers15245784

**Published:** 2023-12-10

**Authors:** Taha Ahmedna, Harmon Khela, Carly Weber-Levine, Tej D. Azad, Christopher M. Jackson, Kathleen Gabrielson, Chetan Bettegowda, Jordina Rincon-Torroella

**Affiliations:** 1 Department of Biology, Johns Hopkins University, Baltimore, MD 21287, USA; 2 Department of Public Health Studies, Johns Hopkins University, Baltimore, MD 21287, USA; 3 Department of Neurosurgery, School of Medicine, Johns Hopkins University, Baltimore, MD 21287, USA; 4 Department of Molecular and Comparative Pathobiology and Oncology, School of Medicine, Johns Hopkins University, Baltimore, MD 21287, USA

**Keywords:** γδ T cells, glioblastoma, immunotherapy, MHC-independent, NKG2D, tumor microenvironment

## Abstract

**Simple Summary:**

Glioblastoma is one of the most aggressive brain tumors, with poor survival and early recurrence rates. The development of effective immunotherapy treatments has been limited by the infiltration-resistant and immunosuppressive tumor microenvironments of these tumors. γδ T cells are unconventional T cells that have the potential to overcome these challenges through their unique recognition of molecular targets on glioblastoma cells. Nonetheless, challenges exist in the utilization of these cells, including their isolation, expansion, and potential for protumor activity. In this review, we discuss the biology of γδ T cells and their role in immunotherapy for glioblastoma and propose several research avenues for future studies.

**Abstract:**

Cell-based immunotherapy for glioblastoma (GBM) encounters major challenges due to the infiltration-resistant and immunosuppressive tumor microenvironment (TME). γδ T cells, unconventional T cells expressing the characteristic γδ T cell receptor, have demonstrated promise in overcoming these challenges, suggesting great immunotherapeutic potential. This review presents the role of γδ T cells in GBM and proposes several research avenues for future studies. Using the PubMed, ScienceDirect, and JSTOR databases, we performed a review of the literature studying the biology of γδ T cells and their role in GBM treatment. We identified 15 studies focused on γδ T cells in human GBM. Infiltrative γδ T cells can incite antitumor immune responses in certain TMEs, though rapid tumor progression and TME hypoxia may impact the extent of tumor suppression. In the studies, available findings have shown both the potential for robust antitumor activity and the risk of protumor activity. While γδ T cells have potential as a therapeutic agent against GBM, the technical challenges of extracting, isolating, and expanding γδ T cells, and the activation of antitumoral versus protumoral cascades, remain barriers to their application. Overcoming these limitations may transform γδ T cells into a promising immunotherapy in GBM.

## 1. Introduction

Glioblastoma (GBM) is among the most aggressive brain tumors, possessing a poor survival rate upon diagnosis and virtually always recurring [1]. The pervasive infiltration of GBM in the brain often precludes complete removal through surgical means. Thus, a wide modality of approaches, including radiation, chemotherapy, targeted therapies, electric fields, and immunotherapy, are used to increase survival [2,3]. GBM immunotherapy has faced severe challenges in successfully reaching and eliminating cancerous cells due to immunosuppression from the GBM tumor microenvironment (TME) and the prevalence of immune escape mechanisms [4,5]. The deep penetration of GBM and the physical limitations imposed by the blood–brain barrier make it difficult for peripheral immune cells to reach the tumors. Those immune cells that do successfully infiltrate tumors are subsequently hindered by the immunosuppressive and hypoxic conditions of the GBM TME [6]. GBM cells can also escape the immune response by shedding existing major histocompatibility complex (MHC) class I proteins, which conventional immune cells depend on for recognition by MHC antigen presentation [4]. Therefore, new strategies of immunotherapy for GBM are needed to address these existing challenges. 

**Figure 1:**
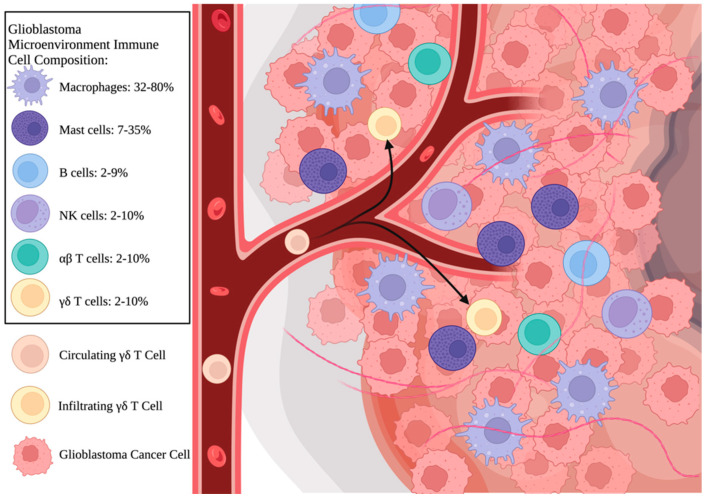
Illustration of γδ T cell recruitment into the GBM microenvironment. The GBM tumor microenvironment is composed of a diverse array of tumor-infiltrating leukocytes (TILs), which are recruited to the tumor in response to tumor-associated antigens. Macrophages make up the bulk (32–80%) of the GBM TIL population, followed by mast cells (7–35%). NK cells and T cells combined comprise 6–30% of TILs, and B cells make up less than 10%. Among infiltrating T cells, the ratio of αβ to γδ T cells is estimated to be approximately equal. Circulating γδ T cells migrate to tumors through TCR-mediated recognition of tumor antigens. Once recruited, the circulating lymphocytes penetrate through the blood vessels and enter the tumors, where they become infiltrating γδ T cells. The percentages used are estimates drawn from reference material published by Lee et al. and are consistent with prior published reports [9,10,11,12,13]. *Created with BioRender.com and published with permission*.

A compelling avenue of immunotherapy utilizes γδ T cells, which do not rely on MHC recognition, to enact immune responses. γδ T cells are unconventional T cells characterized by their expression of the γδ T cell receptor (γδTCR). The novel application of γδ T cells to immunotherapy has shown promise due to their ability to infiltrate a wide variety of tumors with potentially less risk of MHC immune evasion [7]. These unique T cells can commonly be found among tumor-infiltrating leukocytes (TILs) and have exhibited strong natural cytotoxicity towards a variety of solid and circulating cancers, including GBM (Figure 1) [8]. Studying the interaction between the GBM TME and infiltrating γδ T cells can benefit our understanding of GBM pathogenesis, progression, and response to treatment. Moreover, γδ T cells possess great potential as therapeutic agents, and the study of circulating γδ T cells can be utilized in the development of new cell-based therapies. This review examines the role of γδ T cells in GBM, evaluates their utility in cellular-based therapies, and highlights potential future directions (Supplementary Figure S1). 

## 2. Introduction to γδ T Cells

γδ T cells are innate lymphocytes with a TCR composed of γ and δ chains, unlike the αβ chains that comprise most TCRs [14]. They have immense structural and functional heterogeneity and can recognize a wide array of different ligands. Due to the antigenic diversity, γδ T cells employ various TCR repertoires and distinct mechanisms to carry out γδ T cell responses. The γδTCRs expressed on their cellular surfaces can detect and bind antigens in an MHC-unrestricted manner, rendering γδ T cells functionally independent of antigen-presenting cells (macrophages, B cells, dendritic cells), an appealing attribute for immunotherapeutic development and antitumor immunity [14]. 

Variations in the type of TCR Vδ chain of γδ T cells classify them into three major distinct populations, Vδ1, Vδ2, and Vδ3 [15]. The expression of the Vδ2 chain is most prevalent among circulating γδ T cells in human blood, with the Vδ2 identity accounting for 50–95% of peripheral blood γδ T cells and commonly paired with the Vγ9 chain, forming the Vγ9Vδ2 T cell subtype [15]. Vγ9Vδ2 T cells can be activated by phosphoantigens and pyrophosphates emerging from the mevalonate pathway of isoprenoid biosynthesis in eukaryotes [16,17]. 

Activated Vγ9Vδ2 cells can infiltrate the brain parenchyma in response to infection and are thus potent producers of immunostimulatory cytokines such as interferon-γ (IFN-γ) and tumor necrosis factor-α (TNF-α) [14]. These inflammatory cytokines have crucial functions in the immune antitumor response. IFN-γ is a potent activator of macrophages, dendritic cells, and cytotoxic T cells. It exerts antitumor cytotoxicity in tandem with perforin and granzyme and can enhance TNF-mediated apoptosis [18]. TNF-α is a cytokine involved in several signaling cascades that predominantly result in the induction of apoptosis in target cells, but it can also potentiate the activation of cytotoxic T cells [18]. The release of IFN-γ and TNF-α is associated with strong immunological antitumor responses and, as sources of these cytokines, γδ cells are thus important modulators of the immune response to cancer [19].

While there is no known native γδ T cell population in the brain parenchyma, the other two populations of γδ T cells, Vδ1 and Vδ3, typically reside in tissue at mucosal sites. Cells of these subtypes can be found patrolling or residing in the meninges and are involved in the homeostatic maintenance of the brain [20]. They have been shown to promote the response to immune checkpoint blockade in HLA-class-I-negative mismatch-repair-deficient cancers that have the genomic inactivation of β2-microglobulin [21]. γδ T cells execute functions of innate as well as adaptive immunity, since they feature clonally rearranged TCR genes, but do not require antigen presentation or processing to exert effector functions [22].

## 3. Antitumor Activity of γδ T Cells in GBM

**Figure 2:**
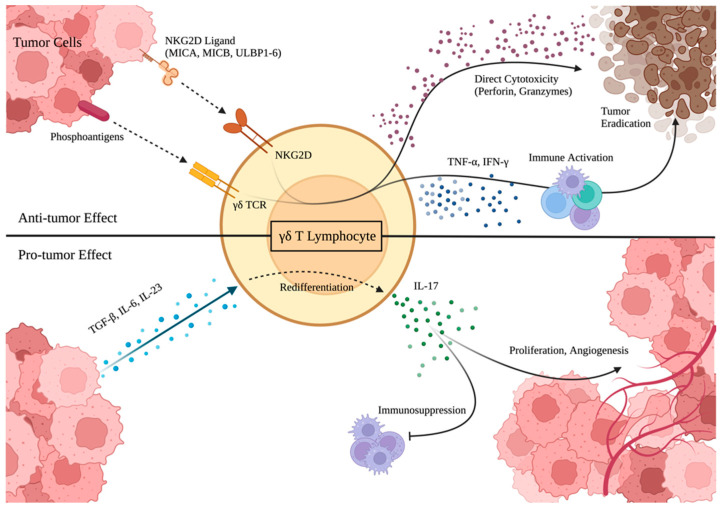
Illustration of antitumor and protumor effects of infiltrating γδ T cells. Infiltrating γδ T cells are thought to display great plasticity and to be heavily influenced by their environment. NKG2D ligands and phosphoantigens in the GBM tumor microenvironment engage with the NKG2D receptor and γδ TCR, respectively (dotted arrows). Antitumor γδ T cells exhibit direct cytotoxicity through the release of perforin and granzymes, which lyse tumor cells. Alternatively, the release of several cytokines (IFN-γ, TNF-α) results in the immune activation of several tumor-infiltrating leukocytes, including NK cells, macrophages, and other T cells. However, the presence of a significant concentration of TGF-β, IL-6, and IL-23 can induce IL-17 production from infiltrating γδ T cells, resulting in protumor activity. IL-17 can directly promote angiogenesis and tumor migration and proliferation while also facilitating an immunosuppressive signal cascade. *Created with BioRender.com and published with permission*.

γδ T cells have demonstrated promising potential as effective immunotherapeutic agents against a variety of brain cancers, including GBM (Table 1). This reactivity is associated with TCR-mediated cytotoxicity coupled with the constitutive expression of natural killer (NK) cell receptors, like NKG2D, on γδ T cells [23,24]. Gliomas express detectable concentrations of ligands that are recognized by the γδ TCR and NKG2D receptors present on γδ T cells [25] (Figure 2). Eight NKG2D ligands (NKG2DL), all found in GBM, are known: two from the group of MHC class I chain-related proteins, MICA and MICB, and six from the UL16-binding protein family, ULBP1-6 [26,27]. One study demonstrated that γδ T cells exposed to mitogen, an NKG2DL, developed lytic activity against both GBM cell lines and patient-derived explants in vitro [28]. Indeed, the increased presence of NKG2DL among in vitro GBM cell lines treated with Temozolomide (TMZ) was linked with increased γδ T cell lysis of the GBM cells when in co-culture, indicating that the expression of NKG2D in γδ T cells contributes to their ability to recognize and lyse tumor cells [24]. 

An important aspect of γδ T cell reactivity towards tumors is the conservation of NKG2D expression among the various subtypes of γδ T cells [23]. Vγ9Vδ2 T cells, expressing NKG2D, can potentially eradicate NKG2DL-presenting GBM cells and, more specifically, mesenchymal GBM cells [39]. In an in vitro study of human primary GBM cultures, γδ T cells were shown to be activated by exposure to GBM and even polarized towards a strong antitumor identity when exposed to the GBM culture supernatant [23]. When co-cultured with human primary GBM cells, such antitumor activity was characterized by the increased production of cytokines, particularly of intracellular perforin, IFN-γ, and TNF-α [37]. This demonstrated the ability of γδ T cells to identify and attack GBM cells. This reactivity towards GBM tumors has been shown to be resilient and effective both naturally and when implemented through adoptive transfer therapies. In murine models, following adoptive transfer, allogeneic human Vγ9Vδ2 T cells were able to survive and pervade within the brain parenchyma for several days and successfully eradicated infiltrative orthotopic primary human GBM cells [8]. Allogenic γδ T cells, when stimulated in vitro and transferred to a murine host, maintained the ability to target and eliminate the human primary GBM cells of the host [35]. Overall, the most effective method of using the natural properties of γδ T cells has been adoptive cell therapy. However, new avenues of γδ T cell therapy have focused on sensitizing γδ T cells to GBM and enhancing their cytotoxicity to specific cancer cell lines.

## 4. Phosphoantigen Stimulation and Combined Therapies

To achieve greater efficacy for clinical use, the natural reactivity of γδ T cells can be enhanced through stimulation with phosphoantigens. Other approaches have also investigated the possible synergistic effect of TMZ in enhancing the targeting of GBM. In in vivo GBM xenografts, the intraperitoneal administration of minodronate, a nitrogen-containing bisphosphonate, induced antitumor effects on GBM tumors and acted synergistically with γδ T cells [34]. Stimulation with the phosphoantigen (E)-4-hydroxy-3-methyl-but-2-enyl pyrophosphate (HMBPP) contributed to γδ T cell differentiation into a T helper 1 (Th1)-like profile when cultured with primary GBM cultures or derived medium [37]. The Th1 profile has been known to have an antitumor response and is associated with the increased production of IFN-γ and TNF-α. In fact, a reliable indicator of the successful response of γδ T cells is IFN-γ expression [19]. Many experiments thus use IFN-γ as an indicator of the magnitude of activity. The finding that phosphoantigen stimulation induced the adoption of a Th1-like profile by γδ T cells alludes to the potential of building larger-scale immune responses towards GBM cells [37]. Additionally, though phosphoantigen stimulation is commonly used to prepare γδ T cells for adoptive transfer therapy, the cytotoxicity induced by lymphocytes can further be enhanced by combining them with chemotherapy or radiation. Studies have demonstrated that TMZ increases NKG2D ligands on GBM cells, increasing their susceptibility to γδ T-cell-mediated clearance [24,25,31]. To overcome the toxic effects of TMZ, Lamb et al. genetically engineered γδ T cells to resist TMZ toxicity by inserting an MGMT transgene, producing the DNA repair enzyme O(6)-alkyguanine DNA alkyltransferase (AGT) [31]. This alteration did not affect the potency of the γδ T cell killing of GBM cells; rather, the TMZ-resistant lymphocytes offered a combinatorial approach to merging chemotherapy with γδ T cell immunotherapy. Additionally, other in vitro investigations have shown that allogeneic human Vγ9Vδ2 T cells exert cytotoxic effects on and spontaneously eliminate GBM cells with a mesenchymal signature. In particular, γδ T cells activated with zoledronate and IL-2 were shown to significantly eradicate cells from three GBM cell lines (U87MG, U138MG, and A172) [32]. Finally, since NKG2D was found to be an integral part of the anti-GBM-induced cytotoxicity by γδ T cells, increasing the GBM cell expression of NKG2DLs would have the effect of further inciting the γδ T cell lysis of tumor cells. Indeed, treating glioma cells with TMZ and/or radiation resulted in the increased surface expression of NKG2DL, which sensitized the GBM cells to cell killing by γδ T cells and precipitated a greater degree of γδ T-cell-induced lysis of GBM cells [26]. In NKG2D-intact mice, γδ T cells produced more IFN-γ upon treatment with TMZ, proving that TMZ treatment facilitated enhanced Vγ9Vδ2 T-cell-mediated GBM cell killing [24].

## 5. Challenges to Clinical Use

Despite its promising potential, there are existing challenges facing the clinical use of γδ T cells in cell-based therapies. First, since γδ T cells account for a minor population of T lymphocytes and there are differences in the cells between humans and mice, translating the results from one species to another is challenging. Though mice and human γδ T cells share several characteristics, evolutionary divergences, especially in the γδ TCR, make direct comparisons difficult [40]. The attested γδ T cell antitumor activity in mice should not be assumed to be retained in humans. Several publications report on the efficacy of human γδ T cells specifically in GBM, a summary of which can be found in Table 1. However, more investigations of human γδ T cells’ anti-GBM properties are necessary to corroborate any murine-based findings [41]. 

Second, most clinical trials of cancer treatment with γδ T cells have used the Vδ2^+^ type, particularly the Vγ9Vδ2 subset, due to their higher abundance relative to other types, leaving the Vδ1 and Vδ3 chains under-investigated [41]. However, since γδ T cells comprise only 1–5% of the T cells in human peripheral blood and the Vγ9Vδ2 subset represent only 5–10% of CD3^+^ type cells, harvesting and expanding γδ T cells cost-efficiently is difficult [42]. Traditionally, γδ T cells are harvested from the population of peripheral blood mononuclear cells (PBMCs) using the Ficoll-Paque separation method. Once harvested, they go through multiple technically challenging isolations for purification from other immune cells, particularly NK cells [43]. While no standard method of γδ T cell isolation and expansion prevails, immunomagnetic cell separation is also commonly used. Prior studies have shown that they can be expanded after isolation [43]. However, the harvesting and purification of γδ T cells remains the most significant technical hurdle for their clinical use [44,45]. 

Finally, the potential protumor properties of γδ T cells present a clear challenge to their therapeutic use. For example, studies have shown that γδ T cells suppress antitumor immune responses in breast cancer and are linked to poor survival in colorectal, breast, and gallbladder cancer [46,47,48]. Studies in brain tumors present contradictory findings, with protumor and antitumor activity both evidenced in different conditions.

## 6. Protumor Activity of γδ T Cells

The potential for protumor activity by certain subtypes of γδ T cells stands as a significant caveat inherent to γδ T cell immunotherapy. While γδ T cells can successfully infiltrate tumors, differences in the composition of the TME may determine whether they execute a pro- or antitumor effect [19]. (Figure 2) If certain TME conditions are present, tumor-infiltrating γδ T cells will initiate a protumor response associated with interleukin-17 (IL-17) production [19]. As a cytokine ordinarily active in pro-inflammatory responses, IL-17 can be produced by CD4^+^ T cells, NK T cells, and γδ T cells [49]. IL-17 has a broad range of functions related to adaptive immunity and mediates several effector cytokine cascades in the immune response to bacterial, fungal, and parasitic pathogens, particularly within mucosal barriers [49]. It has a key role in neutrophil recruitment as well as the direct neutrophil- and macrophage-mediated killing of pathogens and is a prominent potentiator of the inflammatory response [50]. However, the chronic inflammation linked to IL-17 has been shown to exert a pro-tumorigenic effect and contributes to immunosuppression within the TME [47]. In several cancers, IL-17 has been indicted as an inducer of tumor angiogenesis and is generally associated with poor survival [46,48]. In the TME of GBM, some studies indicate that the γδ T cell production of IL-17 can promote tumor growth and survival [19,51].

Within tumors, the emergence of the IL-17 production phenotype is linked to the tumor necrosis factor receptor (TNFR) CD27. CD27^+^ γδ T cells respond to ligands by producing IFN-γ, which is associated with an antitumor effect, whereas CD27^−^ γδ T cells may be responsible for IL-17 production [52]. While this quality is useful for adaptive immune responses, in the context of the TME, it can result in the rapid production of IL-17 and a potential protumor effect. The primary source of protumor IL-17 production within TMEs has not been previously established, but there is evidence that γδ T cells play a role in this phenotype [52]. 

The mechanism of inducing CD27^−^ γδ T cell populations into producing IL-17 involves the engagement of certain ligands of γδTCR and NKG2D and cytokines in the TME, such as IL-1β, IL-6, IL-23, and TGF-β [53,54]. As a pleiotropic cytokine, TGF-β has several immune-related functions, including both pro- and anti-inflammatory effects. It is commonly produced by cancer cells and, in the context of the TME, promotes angiogenesis and facilitates immunosuppression through driving the differentiation of regulatory T cells [55]. TGF-β is also implicated in driving γδ T cells towards IL-17 production, acting concertedly with IL-6 to induce an IL-17-producing identity [56,57]. The pro-inflammatory cytokines IL-1β and IL-23 have major roles in autoimmunity and are key to the development of Th17 cells, which innately produce IL-17, from naïve T cells in response to viral and microbial infection [58]. Studies have shown that IL-23 in combination with IL-1β can induce the constitutive expression of IL-17 in γδ T cells directly or promote their expansion in tandem with TGF-β [57,58]. Thus, it is likely that a combination of these cytokine signals in the TME drives γδ T cells towards IL-17 production and brings about the protumor effect. Although understudied, if γδ T cell IL-17 production persists within GBM, it would be critical to further investigate the specific role of IL-17 in the GBM TME.

Within GBMs, the role of IL-17 has not been thoroughly investigated, with sparse evidence supporting both its pro- and antitumor effects. Some studies have correlated IL-17 production in GBM with enhanced tumor metastasis. Zheng et al. found that U251 and U87MG GBM cells exposed to exogenous IL-17A in vitro exhibited increased aggression and a greater capacity for migration and invasion [59]. The presence of IL-17 increased GBM cell survival and proliferation through PI3K/Akt pathway activation, which is a major regulator of cell survival, migration, and invasion [59]. However, there have been findings that contradict the notion of a universal protumor effect of IL-17 in GBM. In an assessment of GBM tissue resected from 41 patients, increased IL-17 expression was associated with a more favorable prognosis. Patients who had elevated levels of IL-17 expressed in tumors had significantly longer survival than patients with low IL-17 [51]. The correlation between higher IL-17 production and increased survival was consistent with some studies in other cancers, such as ovarian and small cell lung cancers, while others maintained the role of IL-17 as a negative prognostic indicator [60,61,62]. Thus, additional studies are needed to elucidate which conditions render IL-17 a pro- or antitumor agent in GBM.

## 7. Effect of TME Hypoxia on γδ T Cells 

A characteristic feature of GBMs is their immunosuppressive TME, consisting of inhibitory regulators of the immune response and overall hypoxic conditions [63]. Hypoxia is prevalent in many solid malignant tumors and especially in GBM, where vessel occlusion and dysfunctional vascularization lead to significantly reduced oxygen levels from the physiological tissue conditions of 2–9% O_2_ [63,64]. A 2002 study investigating changes in oxygenation within GBMs recorded intratumoral oxygen levels as low as 1.25% and 1.0% O_2_ while awake and asleep, respectively [65]. Such low O_2_ levels within the TME allow for the recruitment of immunosuppressive tumor-associated macrophages (TAMs) and regulatory T cells, which, in hypoxic conditions, suppress T cell responses and negatively regulate their function [66]. 

Oxygen-poor TMEs have been known to inhibit the effectiveness of immune cells, a characteristic observed in γδ T cells [67,68]. The few studies examining the effect of TME hypoxia on γδ T cells have shown that such hypoxia-induced immunosuppression is conserved among them. In vitro studies on oral tumor and breast cancer cell lines showed the diminished cytotoxic capability of γδ T cells in hypoxic conditions [68,69]. One GBM-centered study also investigated the effect of hypoxia on γδ T cells. Park et al. examined the cytotoxicity of γδ T cells in vivo and in vitro under hypoxic conditions in a GL261 orthotopic high-grade glioma (HGG) murine model. They discovered that hypoxia was associated with decreased expression of NKG2D in γδ T cells, an increased presence of hypoxia-inducible factor 1-α (HIF1α), and decreased overall cytotoxic activity. Conversely, when hypoxia was alleviated through the administration of metformin and inhibition of HIF1α, NKG2D expression was enhanced, and the antitumor response returned [36]. These results indicate that hypoxia may inhibit the antitumor effect of γδ T cells in GBM by driving a reduction in the expression of NKG2D as one of the mechanisms [36]. Additionally, some studies have linked the activation of HIF1α with IL-17 production related to inflammatory immune responses, indicating that tumor hypoxia may contribute to IL-17 production and the associated protumor effect [70]. However, the relationship between HIF1α and IL-17 has not been extensively examined, and thus no definitive conclusion can be drawn. These findings represent only a few studies examining the relationship of γδ T cell cytotoxicity and hypoxia in GBM. The mechanistic influence of hypoxia on γδ T cells is a critical avenue to investigate in future studies.

## 8. Future Directions

The efficacy of γδ T cells against GBM gives them potential as an immunotherapeutic approach. Their MHC independence and the presence of combined NKG2D and γδ TCR-facilitated tumor recognition endows the γδ T cells with natural antitumor effects and the potential to overcome key immune evasion mechanisms of GBM [14,24]. Such qualities can be harnessed and amplified through phosphoantigen stimulation and combined with chemotherapy approaches, making for an attractive clinical application [37,38]. However, several gaps in our understanding must be filled before γδ T cells can be used as immunotherapeutic agents in GBM. First, the risk of a protumor effect stands as a fundamental caveat for γδ T cell immunotherapy that requires further exploration (Figure 2). The relatively few studies conducted on IL-17 in γδ T cells and its association with different outcomes are contradictory, with studies reporting both pro- and antitumor effects [51,59]. It is likely that different TME conditions influence the protumor potential of γδ T cells [19]. Therefore, additional studies analyzing γδ T cells in co-culture with GBM will further elucidate the pathology of the protumor phenotype. It is also particularly important for in vivo studies to investigate the conditions and TME components, including hypoxia, that may polarize γδ T cells towards a protumor identity [60,70]. Understanding the main players influencing the protumor potential of γδ T cells is necessary to ensure optimal safety for clinical usage. Further, due to the differences between γδ T cells in humans and mice, any results found in murine models must be replicated in humans [40].

Beyond in vivo and in vitro studies, clinical investigations are warranted to clarify the role of γδ T cells within GBM patients. γδ T cell infiltration of many cancer types, including GBM, generally confers a positive prognosis [19,71]. There is also evidence, however, that in progressed tumor conditions, their presence can be a negative survival indicator [19]. This question could be answered by harvesting pathological tissue, performing single-cell RNA sequencing to identify γδ T cells, and identifying the relationship between survival and the magnitude of γδ T cell activity. Large-scale databases, such as the TCGA, may also be useful resources for studying the clinical impact of γδ T cell infiltration retrospectively. 

The final barriers to the clinical use of γδ T cells are harvesting, purifying, and expanding the cells. The low population of γδ T cells in the blood and the difficulty of purifying them from other lymphocytes make the process cost-inefficient for production at the scale necessary for clinical implementation [42,43,44,45]. The refinement of existing techniques or the discovery of new ones could help to alleviate this limitation. Once the production of γδ T cells is more cost-efficient, then they could be reliably used in GBM immunotherapy research. 

## 9. Conclusions

With γδ T cells’ unique ability to recognize antigens independent of MHCs and infiltrate tumors, they have incredible potential to serve as a new treatment strategy for patients with a variety of cancers, especially GBM. In this review, we presented an overview of the biology of γδ T cells, as well as discussing the existing challenges to their clinical use. We described the mechanisms of their potential pro- and antitumor effects. We highlighted several key remaining questions and the potential research projects to address them. Ultimately, remedying these shortcomings in our knowledge of γδ T cells and their immunotherapeutic potential will pave the way for their clinical use against GBM.

## Figures and Tables

**Table 1:** Overview of relevant γδ T cell studies in GBM (*n* = 15). [8,13,23,24,28,29,30,31,32,33,34,35,36,37,38].

Authors, Year	Methods	Cell Lines	Questions Addressed	Results
Bryant et al., 2009 [28]	In vitro	U251MG, D54MG, U373MG, U87MG, normal human astrocyte cultures and primary GBM cultures from operative specimens	What is the innate role of γδ T cells in the immune response to GBM and how potent are they therapeutically against GBM cell lines and primary GBM cultures?	Before tumor resection, the number of γδ T cells was significantly lower in GBM patients compared to normal numbers and the mitogenic proliferative response was substantially diminished, indicating γδ T cell immunosuppression concurrent with tumor growth. Expanded and activated γδ T cells, both those sourced from healthy controls and those from patients, killed cells from existing GBM cell lines as well as patient-derived GBM tumor explants in culture. Mitogen-stimulated γδ T cells did not kill primary human astrocytes and had more effective lytic activity compared to αβ T cells in the same conditions.
Bryant et al., 2011 [29]	In vivo	U251MG, U251^ffluc^	What is the immunotherapeutic potential of γδ T cells following in vivo injection? How effectively do γδ T cells migrate to and infiltrate GBM tumor xenografts in immunodeficient mice?	γδ T cells expanded and activated ex vivo demonstrated robust antitumor effects on GBM cells in vitro. After the stereotactic intracranial implantation of GBM tumor cells into athymic nude mice, the tumor masses of mice treated with γδ T cells showed markedly slower progression. In all groups treated with γδ T cells, median survival was consistently improved compared to control mice, potentially constituting a new, effective immunotherapeutic approach.
Cimini et al., 2011 [30]	In vitro	T70, U251, U373	Does bisphosphonate treatment sensitize glioma cells to lysis by γδ T cells expanded in vitro?	Human Vδ2 T cells were expanded in vitro by phosphoantigen stimulation. Vδ2 T cells differentiated into effector/memory cells when co-cultured with GBM cells and killed these cells by releasing perforin. Zoledronic acid treatment induced the dose-dependent apoptosis of T70 glioma cells and activated Vδ2 T cells and significantly enhanced their antitumor cytotoxicity against GBM cells.
Lamb et al., 2013 [31]	In vitro	U87, U373, SNB-19	What are the effects of combining TMZ-mediated cytotoxicity and γδ T cell immunotherapy for GBM?	When exposed to TMZ, resistant GBM cells exhibited increased expression of NKG2D ligands. γδ T cells, normally susceptible to TMZ-mediated cytotoxicity, were genetically modified via a methylguanine DNA methyltransferase (MGMT) transgene encoding AGT to feature TMZ resistance. Conferring TMZ resistance did not, however, weaken their cytotoxicity against GBM cells in vitro. When TMZ-resistant GBM cell lines were treated with TMZ and exposed to γδ T cells, the TMZ-resistant γδ T cells exhibited a more potent cytotoxic effect compared to unaltered lymphocytes, indicating that exposure to TMZ resulted in more receptors for γδ T-cell-mediated lysis.
Nakazawa et al., 2014 [32]	In vitro	U87MG, U138MG, A172	How potent is γδ T cell immunotherapy for GBM patients and how can the γδ T-cell-mediated killing of tumors be enhanced?	γδ T cells exhibited lower inherent cytotoxicity towards GBM cells as compared to other cell lines. Of the GBM cell lines treated with γδ T cells, 32% of U87MG, 15% of U138MG, and 1% of A172 were killed, compared to 50% killing of leukemia cell line K562. Sensitizing GBM cell lines with zoledronate significantly increased γδ T-cell-mediated killing, with 86% of U87MG, 43% of U138MG, 67% of A172 cells, and 55% of K562 cells killed in the same conditions, indicating that pre-treatment with zoledronate significantly improved the cytotoxic killing of GBM. However, an anti-TCR antibody inhibited this zolendronate-enhanced killing, indicating that the γδ T cell receptor is crucial for effective cytotoxicity.
Beck et al., 2015 [33]	In vivo	GL261	What are the properties of γδ T cell activity against high-grade glioma in fully immunocompetent mouse models?	In mice engrafted with intracranial gliomas, the circulating γδ T cell counts increased 10–12 days after tumor implantation but declined precipitously at the end stage. Overall, 12% of the total γδ T cells produced IFN-γ, while none produced IL-17 or IL-4, despite the presence of TGF-β, an immunosuppressive cytokine. Glioma production of TGF-β was increased when exposed to T-cell-derived media, suggesting that immunosuppression increases concurrently with immune activation. Tumor progression and survival advantage was nearly the same in γδ T cell KO mice and in wild-type mice, suggesting an unsustainable and insufficient cytotoxic effect of γδ T cells in vivo. The combined results indicate that the immunosuppressive TME protects tumors from robust γδ T-cell-mediated killing, with an immune response characterized by the early proliferation of γδ T cells with an initial tumor reduction followed by rapid T cell deletion and tumor resurgence.
Chitadze et al., 2016 [24]	In vitro	A172, T98G, U87MG, U251MG	What is the effect of metalloprotease inhibitors and TMZ on NKG2DL expression in human GBM cell lines?	GBM cells expressed many NKG2DLs (MICA, MICB, ULBP1, ULBP2), yet ULBP2 was preferentially secreted in an ADAM10/17-dependent fashion. Treatment with TMZ upregulated ULBP2 cell surface expression in GBM cell lines and sensitized GBM cells to γδ T cell killing. Stimulating γδ T cells with pyrophosphate antigens (BrHPP) substantially enhanced GBM cell lysis.
Jarry et al., 2016 [8]	In vivo	U87MG, GBM-10 (primary GBM cell culture)	What is the efficacy of stereotactic γδ T cell injection immunotherapy in murine models of human GBM tumors and in primary GBM cells?	Human γδ T cells of the subtype Vγ9Vδ2 were stereotacically injected into the brain parenchyma of mice. The T cells survived and patrolled for several days and successfully eliminated infiltrative GBM primary cells in vivo. Stereotactic γδ T cell injection eradicated zoledronate-sensitized U87MG and GBM-10 cells, thereby improving survival compared to the absence of zoledronate sensitization. The results demonstrate that the stereotaxic injection of allogeneic human Vγ9Vδ2 cells in vivo, coupled with zoledronate treatment, can efficiently kill primary human GBM tumors, creating potential for further optimization.
Nakazawa et al., 2016 [34]	In vitro and in vivo	U87MG, U138MG	What are the synergistic antitumor effects, if any, of minodronate on the γδ T-cell-mediated killing of GBM cells in vitro and in vivo?	Minodronate (MDA) administered in vitro to GBM cell lines directly inhibited cell growth in a dose-dependent fashion. When administered alone, γδ T cells did not inhibit cell growth. However, GBM cell growth was inhibited to a greater degree when treated with both MDA and γδ T cells in co-culture than with MDA treatment alone. MDA first exposed GBM cells to γδ-TCR recognition, and the subsequent release of granzyme B and TNF-α, inducing GBM apoptosis. Intraperitoneal administration of MDA and human γδ T cells in immunocompromised mice resulted in antitumor cytotoxicity, demonstrating for the first time the in vivo antitumor effects of MDA when in the presence of γδ T cells.
Joalland et al., 2018 [35]	In vivo	GBM-1 (primary human GBM cell culture), U87MG	What effect does IL-21 sensitization have on the cytolytic activity of γδ T cells in GBM cells?	The Vγ9Vδ2 subtype of γδ T cells naturally eliminated GBM-1 cells in vitro using perforin/granzyme-mediated cytotoxicity. Human Vγ9Vδ2 T cells pre-sensitized with IL-21 exhibited increased cytolytic activity against GBM-1 and U87 cells compared to non-sensitized T cells. Mean survival of mice with orthotopically implanted GBM-1 increased from 41 to 66 days after treatment with IL-21-sensitized Vγ9Vδ2 T cells that were stereotactically injected.
Lee et al., 2019 [13]	In vitro	Primary GBM cultures from operative specimens	What characterizes the identity of GBM-infiltrating γδ T cells? How do infiltrating γδ T cells interact with the GBM TME?	γδ T cells derived from GBM patient tumors and blood displayed environment-specific differences in their TCR repertoire. Tumor-infiltrating Vγ9Vδ2 T cells specific to GBM were the most abundant subtype among both tumor-infiltrating and blood-circulating γδ T cells. However, tumor-infiltrating Vγ9Vδ2 T cells used non-canonical Vγ9Jγ2 sequences in their γδ TCR, whereas blood Vγ9Vδ2 T cells had canonical Vγ9JγP sequences. Within the TME, immune-related gene expression analysis revealed that infiltrating Vγ9Vδ2 T cells exhibited gene expression signatures similar to M1 macrophages or Th1 cells and had activity strongly associated with cytotoxicity.
Chauvin et al., 2019 [23]	In vitro and in vivo	GBM-1, GBM-10, human primary GBM cultures from surgical specimens	What are the different immuno-reactivities of Vγ9Vδ2 T cells against various patient-derived human GBM cells?	The Vγ9Vδ2 T cell subtype spontaneously reacted to kill mesenchymal GBM-1 cells in vitro but did not spontaneously kill classical, neural, or proneural (CNP) GBM-10 cells after 4 h of co-culture. Killing of CNP cells was achieved after treatment with NBP but killing of mesenchymal cells did not require pre-treatment. Elimination of mesenchymal GBM cells depended on TCR engagement and NKG2D signaling. NKG2D had increased expression in mesenchymal GBM-reactive Vγ9Vδ2 T cells and the NKG2D pathway was found to regulate cytolytic activity in vivo.
Park et al., 2021 [36]	In vitro and in vivo	GL261, U87MG	How does tumor hypoxia affect γδ T cell antitumor activity?	Patient-derived brain tumor-infiltrating lymphocytes experienced hypoxia within the glioma TME, resulting in repression of antitumor immunity. Tumor-infiltrating leukocyte (TIL) clusters that expressed hypoxia-inducible factor 1-α also showed signs of apoptosis and had weak antitumor effect. Hypoxia was found to inhibit γδ T cell NKG2D through the PKA pathway, thereby reducing antitumor activity. When GL261 cells, which consume oxygen at a high rate, were treated with metformin, which inhibits respiration, the present infiltrating γδ T cells exhibited increased antitumor activity. Alleviation of hypoxia was thus associated with a stronger antitumor response.
Rosso et al., 2021 [37]	In vitro	U251, U373, human primary GBM cultures from surgical specimens	How do phosphoantigen-stimulated γδ T cells respond to human GBM cells in vitro?	Phosphoantigen-stimulated γδ T cells adopted a Th1-like profile when co-cultured with GBM cells, exhibiting increased antitumor activity. HMBPP-stimulated Vγ9Vδ2 T cells were co-cultured with U251 and U373 or GBM-soluble factors. These cells exhibited increased production of intracellular perforin, IFN-γ, and TNF-α. HMBPP-stimulated Vγ9Vδ2 T cells incubated with patient-derived GBM cells also showed increased production of CD68, IFN-γ, and TNF-α, demonstrating activation of a strong Th1-like immune response. Overall survival of GBM patients improved with the increased presence of Vγ9Vδ2 T cells in their tumors.
Lamb et al., 2021 [38]	In vitro and in vivo	Patient-derived tumor xenografts	Do TMZ-resistant γδ T cells exhibit improved survival and efficacy against high-grade gliomas under therapeutic concentrations of TMZ?	γδ T cells with a MGMT transgene were resistant to TMZ, which is normally lymphodepleting. γδ T cell treatment without TMZ and TMZ treatment alone were both less effective against PDX tumors and did not improve survival outcomes significantly. Mice treated with both intracranially administered MGMT-modified γδ T cells and intraperitoneal TMZ, however, exhibited increased median survival compared to mice with single agents. These results provide evidence for the potential of combined therapeutic approaches.

GBM: glioblastoma; TMZ: temozolomide; MGMT: methylguanine DNA methyltransferase; KO: knockout; MDA: minodronate; TCR: T cell receptor; TME: tumor microenvironment; TIL: tumor-infiltrating leukocyte; HMBPP: (E)-4-hydroxy-3-methyl-but-2-enyl pyrophosphate.

## Data Availability

No new data were created in this review.

## Supplementary Materials

The following supporting information can be downloaded at: https://www.mdpi.com/article/10.3390/cancers15245784/s1, Figure S1. Using the databases PubMed, ScienceDirect, and JSTOR, authors searched for published studies on γδ T cells over the period of 1995–2022. The searches were conducted using several terms comprised of combinations of the following: γδ T cells, glioblastoma, GBM, hypoxia, TME, phosphoantigens, IL-17, protumor, antitumor, infiltration, animal models, NKG2D, cytotoxicity, immunotherapy, preclinical models, and clinical trials. Publications were collected and sorted with emphasis put on the recency of studies as well as relevance to glioblastoma immunotherapy. Each article’s reference section was also searched to screen for additional studies. After collecting and reviewing an initial population of publications, subsequent searches were conducted to flesh out specific sections of the review, including the protumor effect, tumor microenvironment, and hypoxia sections. For example, when drafting the ‘Effect of TME Hypoxia on γδ T Cells’ section, the search terms inputted were γδ T cells:TME:hypoxia, and/or γδ T cells:hypoxia.
