# Supplementary material for: The Role of γδ T-Lymphocytes in Glioblastoma: Current Trends and Future Directions
Source: Cancers (Basel). 2023 Dec 10;15(24):5784. doi: 10.3390/cancers15245784 (PMC10741533; doi:10.3390/cancers15245784)
Supplement: Supplementary file 1 [file cancers-15-05784-s001.zip › cancers-2722175-supplementary.pdf]

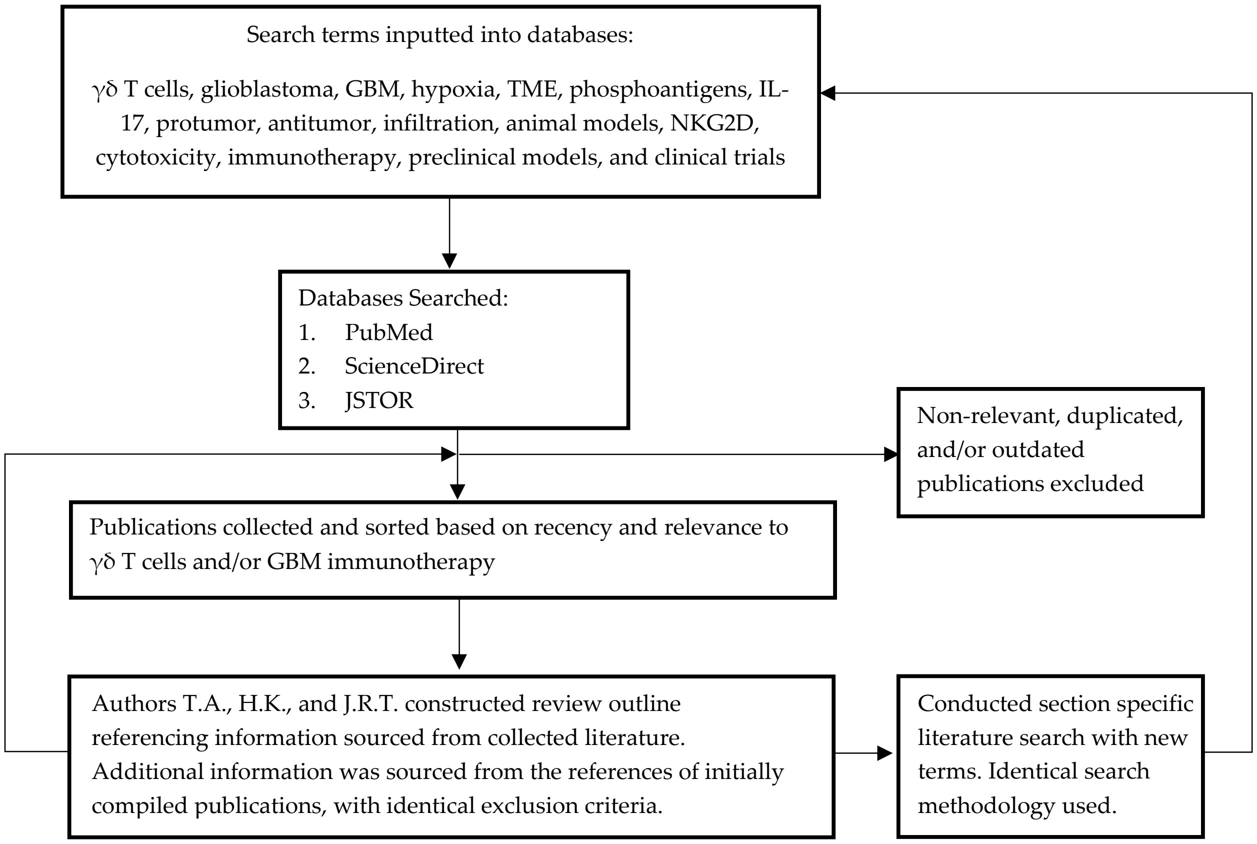

Supplementary Figure S1. Using the databases PubMed, ScienceDirect, and JSTOR, authors searched for published studies on γδ T cells over the period of 1995-2022. The searches were conducted using several terms comprised of combinations of the following: γδ T cells, glioblastoma, GBM, hypoxia, TME, phosphoantigens, IL-17, protumor, antitumor, infiltration, animal models, NKG2D, cytotoxicity, immunotherapy, preclinical models, and clinical trials. Publications were collected and sorted with emphasis put on the recency of studies as well as relevance to glioblastoma immunotherapy. Each article reference section was also searched to screen for additional studies. After collecting and reviewing an initial population of publications, subsequent searches were conducted to flesh out specific sections of the review, including the protumor effect, tumor microenvironment, and hypoxia sections. For example, when drafting the 'Effect of TME Hypoxia on γδ T Cells' section, the search terms input were γδ T cells:TME:hypoxia, and/or γδ T cells:hypoxia.
